# Diagnostic accuracy of the response to the brief tachycardia provoked by standing in children suspected for long QT syndrome

**DOI:** 10.1016/j.hroo.2021.03.005

**Published:** 2021-03-13

**Authors:** Arja S. Vink, Ben J.M. Hermans, Joana Pimenta, Puck J. Peltenburg, Luc H.P.M. Filippini, Nynke Hofman, Sally-Ann B. Clur, Nico A. Blom, Arthur A.M. Wilde, Tammo Delhaas, Pieter G. Postema

**Affiliations:** ∗ Department of Cardiology, Amsterdam UMC, University of Amsterdam, Heart Center, Amsterdam, The Netherlands; † Department of Pediatric Cardiology, Emma Children’s Hospital, Amsterdam UMC, University of Amsterdam, Amsterdam, The Netherlands; ‡ Department of Biomedical Engineering, Maastricht University, Maastricht, The Netherlands; § Cardiovascular Research Institute Maastricht (CARIM), Maastricht University, Maastricht, The Netherlands; ‖ Department of Pediatric Cardiology, Centro Hospitalar de São João, Porto, Portugal; ¶ Department of Pediatric Cardiology, Juliana Children’s Hospital, The Hague, The Netherlands; # Department of Pediatric Cardiology, Leiden University Medical Center, Leiden, The Netherlands

**Keywords:** Children, ECG, LQTS, QTc, QT interval

## Abstract

**Background:**

Adult long QT syndrome (LQTS) patients have inadequate corrected QT interval (QTc) shortening and an abnormal T-wave response to the sudden heart rate acceleration provoked by standing. In adults, this knowledge can be used to aid an LQTS diagnosis and, possibly, for risk stratification. However, data on the diagnostic value of the standing test in children are currently limited.

**Objective:**

To determine the potential value of the standing test to aid LQTS diagnostics in children.

**Methods:**

In a prospective cohort including children (≤18 years) who had a standing test, comprehensive analyses were performed including manual and automated QT interval assessments and determination of T-wave morphology changes.

**Results:**

We included 47 LQTS children and 86 control children. At baseline, the QTc that identified LQTS children with a 90% sensitivity was 435 ms, which yielded a 65% specificity. A QTc ≥ 490 ms after standing only slightly increased sensitivity (91%, 95% confidence interval [CI]: 80%–98%) and slightly decreased specificity (58%, 95% CI: 47%–70%). Sensitivity increased slightly more when T-wave abnormalities were present (94%, 95% CI: 82%–99%; specificity 53%, 95% CI: 42%–65%). When a baseline QTc ≥ 440 ms was accompanied by a QTc ≥ 490 ms and T-wave abnormalities after standing, sensitivity further increased (96%, 95% CI: 85%–99%) at the expense of a further specificity decrease (41%, 95% CI: 30%–52%). Beat-to-beat analysis showed that 30 seconds after standing, LQTS children had a greater increase in heart rate compared to controls, which was more evidently present in LQTS boys and LQTS type 1 children.

**Conclusion:**

In children, the standing test has limited additive diagnostic value for LQTS over a baseline electrocardiogram, while T-wave abnormalities after standing also have limited additional value. The standing test for LQTS should only be used with caution in children.

**Key Findings**

▪ Long QT syndrome (LQTS) can be a challenging diagnosis when based on corrected QT interval (QTc) prolongation, the presence of clinical and other LQTS-associated features, or a confirmed pathogenic genetic variant alone. As a consequence, additional tests have been developed to improve diagnostic accuracy.
▪ In adult LQTS patients an inadequate QTc shortening and abnormal T-wave response to the sudden heart rate acceleration provoked by standing was found. In adults this knowledge base can be used to aid an LQTS diagnosis and, possibly, for risk stratification. However, data on the diagnostic value of the standing test in children are currently limited.
▪ We show that in our prospective cohort of children suspected for LQTS, an LQTS diagnosis can be based on a QTc during QT stretching (ie, at the incidence where the T-wave end is closest to the next P wave) of ≥490 ms with a high sensitivity and acceptable specificity, and that the diagnosis can be made with more confidence when QTc prolongation is accompanied by T-wave abnormalities (ie, *notched*, *biphasic*, and *flat* T waves).
▪ However, in general, the standing test had only slight additional value as a screening test for LQTS or in the discrimination between (borderline) LQTS patients and healthy children when compared to a QTc of 440 ms on a standard resting electrocardiogram.
▪ Beat-to-beat analysis showed that 30 seconds after standing, LQTS children had a greater increase in heart rate compared to controls, which was more evidently present in LQTS boys and LQTS type 1 children, supporting a potential value in analyzing beat-to-beat heart rate and QT interval dynamics during the standing test for risk stratification of LQTS children.
▪ Diagnosing or refuting LQTS can be a delicate issue, especially in children. Owing to the difficulties apparently associated with interpreting a standing test in children, we would advocate its current use in expert centers to be able to gain more insights before more widespread use.

## Introduction

Congenital long QT syndrome (LQTS) is an inherited cardiac arrhythmia disorder associated with malignant ventricular arrhythmias, especially in the young. The hallmark of an LQTS diagnosis is a QT interval prolongation corrected for heart rate (QTc) on a 12-lead electrocardiography (ECG) at rest.^1^ Besides QTc prolongation, LQTS can also be diagnosed by the presence of clinical and other LQTS-associated features or a confirmed pathogenic genetic variant.^1^ These 3 elements in an LQTS diagnosis are hampered by clinical challenges. LQTS patients can have a borderline prolonged or even normal resting QTc,^2^ which implies a considerable QTc overlap between affected and unaffected individuals.^3^ Furthermore, interpretation of symptoms as either benign or malignant can be difficult.^4^^,^^5^ Additionally, distinguishing pathogenic variants from innocuous rare variants can be very complex, especially in the current era of DNA panels and whole-genome sequencing,^6^ while in one-fourth of clinically diagnosed LQTS patients no LQTS mutation is uncovered.^7^

As a consequence, diagnosing LQTS remains challenging and additional tests have been developed to improve diagnostic accuracy. This includes QTc measurements during the recovery phase of exercise^8^^,^^9^ and during epinephrine infusion.^10^^,^^11^ In addition, a “standing test” was developed that showed that adult LQTS patients with intermediate QTc at baseline had an impaired QT interval shortening in response to the brief tachycardia provoked by standing compared to controls.^12^ Moreover, their QTc remained prolonged even after the heart rate returned to baseline conditions,^13^ while simultaneously, abnormal T waves were observed after standing, all with added value for diagnosing LQTS.^14^

Because LQTS can result in severe arrhythmic events in children,^15^ diagnostic tests to evaluate repolarization reserve in children in addition to a resting ECG would be helpful. Moreover, (near) syncope in children—particularly peripuberty—is a rather often-occurring event.^16^ Although vasovagal syncope is its dominant cause (which can also occur in LQTS children), many children are referred for further analyses to exclude LQTS. In this respect, it is important to note that children have higher heart rates during resting conditions and more pronounced reflex tachycardia compared to adults.^17^ In healthy children, the QTc prolongation after standing is also more pronounced than in healthy adults.^18^ Therefore, using adult cut-off values for children may yield false-positive results with the risk of an incorrect LQTS diagnosis and overtreatment. Furthermore, there are currently no data on T-wave morphology changes provoked by standing in children. Here we aimed to evaluate the diagnostic value of the standing test for an LQTS diagnosis in children. In particular, we recapitulated all the methods previously shown in separate papers to be of diagnostic value in LQTS adults.^12^, ^13^, ^14^

## Methods

We performed a prospective cohort study from January 2009 until September 2018. All children aged ≤18 years with an available standing test in the Amsterdam UMC, The Netherlands, were included. The children received a standing test as part of regular care either (1) for family screening in case of familial LQTS or sudden cardiac death in the family, or (2) because of symptoms, often in combination with a prolonged or high-normal QTc.

The study was approved by the Academic Medical Center Review Board and informed consent of the subjects was waived as this study used data from regular care. The research reported in this paper adhered to Helsinki Declaration as revised in 2013.

### Data collection and measurements

#### ECGs and additional data

In all controls (healthy children after evaluation or genotype-negative family members of genotype-positive LQTS patients) and confirmed LQTS children (including a pathogenic variant in *KCNQ1*, *KCNH2*, or *SCN5A*), the standing test ECG was evaluated. The standing test was performed as described previously.^12^, ^13^, ^14^ In brief, children rested supine for several minutes before a continuous 5-minute ECG-recording was started where the children remained supine for 2 minutes and were then asked to stand up and stay standing for the remaining minutes. The original study of Viskin and colleagues^12^ initially used a longer standing phase of 5 minutes. However, from data of that same group we have learned that the heart rate returns to baseline at approximately 30 seconds after standing. Therefore, 3 minutes is sufficient for measurements in steady state and this duration was also more preferable for the children.

As this was a prospective cohort study including standing tests as an initial evaluation tool in addition to a standard ECG, medical history, etc, many children ultimately did *not* receive an LQTS diagnosis. The results of these children, which also include *possible LQTS* (prolonged QTc and/or positive epinephrine test without a confirmed pathogenic variant, family members of genotype-elusive LQTS patients, suspected family history for LQTS) and o*ther children* (screening for aborted cardiac arrest or sudden cardiac death in the family, idiopathic ventricular fibrillation, cardiomyopathies, polymorphisms, Brugada syndrome) are described in Supplemental Table S1.

#### Manual measurements

All standing test ECGs, paper or digitally obtained, were manually analyzed, consistent with previous studies.^12^, ^13^, ^14^ The first 5 seconds after standing were excluded, as artefacts prohibited QT interval analysis. One reader (S.V.), blinded to patient characteristics, measured the QT interval and the preceding R-R interval at 4 periods: (1) *baseline*; supine before standing where the R-R interval corresponds with the longest R-R interval after standing, (2) *maximal tachycardia*; maximal sinus rate in response to standing, (3) *maximal QT stretching*; after standing at the point the T wave approaches nearest to the subsequent P wave, and (4) *return to baseline*; maximal sinus bradycardia while standing. In order to find matching R-R intervals in supine and standing position, the complex with the longest R-R interval within 30 seconds *after* standing (period 4) was used to find a corresponding R-R interval (± 40 ms) for the baseline measurements (period 1). This maneuver precludes excluding period 4 when the heart rate after standing did not return to the initially selected baseline conditions and is a slight deviation of protocol compared to previous studies.^12^^,^^13^

At all periods, the QT interval was determined using the tangent method^3^ and was corrected for heart rate using Bazett.^19^ All QT intervals were measured in 1 lead, preferably in lead II or V_5_.

T-wave morphology assessment was performed at the 4 periods in 4 different lead groups—(1) II, III, and aVF; (2) V_1_–V_3_; (3) V_4_–V_6_; and (4) I and aVL—and was classified as described previously^14^ (Supplemental Figure S1).

#### Automated measurements

To study the dynamic response of the QT interval to the abrupt change in heart rate in more detail, all digitally available standing tests were analyzed beat-to-beat using custom-made software in MATLAB (2018a; MathWorks, Natick, MA). R peaks, QRS onset, and T-wave end were detected using our previously described QT interval algorithm, modified to be used for a single ECG lead to make it more consistent to the manual assessment.^20^ To compare automated measurements to manual measurements, single complexes were chosen based on the same definitions as the manual measurements: (1) baseline, (2) maximal tachycardia, (3) maximal QT stretching, and (4) return to baseline. Furthermore, a moving average filter with a 15-second window with 5-second overlap was applied to the beat-to-beat QT and R-R intervals for every subject. Then, the median (as well as the first and third quartiles) dynamic behaviors of these moving average intervals were calculated.

### Statistical analysis

All data were analyzed with R version 3.4.3 (The Foundation for Statistical Computing, Vienna, Austria). Baseline and ECG characteristics are presented as numbers (percentage, %) for categorical variables and mean (± standard deviation) or median (interquartiles) for continuous variables, stratified by group. Differences between groups were tested using a χ^2^ test for categorical variables and a *t* test or Mann-Whitney *U* test for continuous variables as appropriate.

To test the *diagnostic value* of the standing test for an LQTS diagnosis, receiver operating characteristic (ROC) curve analyses were used to calculate the area under the curve (AUC) and to evaluate the specificity at a predefined sensitivity of 90% (similar to earlier studies).^12^^,^^13^ DeLong’s method^21^ was used to calculate the 95% confidence interval (CI) around the AUC and to compare ROC curves. A logistic-regression analysis was used to determine whether T-wave morphology changes add to diagnostic value by establishing odds ratios.

*Sensitivity analyses* were performed by excluding (1) all children using beta-blocker therapy and (2) all LQTS children with obvious QTc prolongation (≥480 ms)^3^ at baseline, because additional tests can be considered superfluous for these individuals.

Sampling uncertainty was quantified with 95% CI and *P* values < .05 were considered statistically significant.

## Results

Table 1 shows the baseline characteristics of the included 86 controls and 47 LQTS children (26 LQTS type 1, 19 LQTS type 2, 2 LQTS type 3). Both groups were of similar age and showed no statistical difference as to sex (*P* = .630), reason for genetic testing (*P* = .079), or symptoms (*P* = 1.000). There was a difference in the number of children that were on beta-blockers (*P* = .003). There was one control individual on beta-blockers for hypertension.

**Table 1:** Baseline characteristics and manual electrocardiogram measurements

	Control n=86	LQTS n=47	P value
*Age, years*	10 (7–14)	12 (8–15)	1.000
*Girls*	39 (45%)	29 (62%)	.630
*Presentation*			.079
Family screening	47 (55%)	38 (81%)	
Family SCD	8 (9%)	0 (0%)	
Near-drowning/OHCA/ACA	1 (1%)	0 (0%)	
Other	30 (35%)	9 (19%)	
*Symptomatic at presentation*	1 (1%)	3 (6%)	1.000
*BB therapy*	1 (1%)	9 (19%)	.003
*Supine position*†			
HR_baseline_, bpm	81 (± 15)	73 (± 16)	.062
QT_baseline_, ms	367 (± 34)	429 (± 58)	**<.001**
QTc_baseline_, ms	421 (± 29)	466 (± 36)	**<.001**
*Standing position*†			
HR_maxHR_, *bpm*	112 (± 15)	100 (± 17)	**<.001**
QT_maxHR_, ms	360 (± 34)	421 (± 60)	**<.001**
QTc_maxHR_, ms	489 (± 37)	537 (± 51)	**<.001**
HR_stretch_, *bpm*	110 (± 15)	99 (± 17)	**.001**
QT_stretch_, ms	363 (± 36)	429 (± 62)	**<.001**
QTc_stretch_, ms	489 (± 42)	544 (± 56)	**<.001**
QT_return_, ms	371 (± 39)	450 (± 74)	**<.001**
QTc_return_, ms	429 (± 38)	492 (± 60)	**<.001**
*Response to standing*†			
Time to maximal tachycardia, s	11 (9–14)	11 (10–13)	1.000
Time to maximal QT stretching, s	11 (9–14)	10 (9–12)	1.000
Time to return to baseline, s	21 (18–27)	20 (19–29)	1.000
ΔHR during maximal tachycardia, bpm	32 (± 11)	27 (± 9)	.031
ΔQT during maximal tachycardia, ms	-9 (± 22)	-8 (± 30)	1.000
ΔQTc during maximal tachycardia, ms	67 (± 41)	71 (± 47)	1.000
ΔHR during maximal QT stretching, bpm	31 (± 11)	26 (± 9)	.038
ΔQT during maximal QT stretching, ms	-7 (± 21)	0 (± 39)	1.000
ΔQTc during maximal QT stretching, ms	67 (± 43)	78 (± 54)	1.000
ΔQT upon return to baseline HR, ms	5 (± 28)	22 (± 47)	.140
ΔQTc upon return to baseline HR, ms	8 (± 31)	26 (± 50)	.120

ACA = aborted cardiac arrest; BB = beta-blocker; bpm = beats per minute; HR = heart rate; OHCA = out-of-hospital cardiac arrest; QTc = corrected QT interval; SCD = sudden cardiac death.

† *P* value < .002 is statistically significant and presented in bold.

### Manual measurements

Table 1 shows the results for the manual measurements. As expected, LQTS children had a longer QT interval and QTc at baseline compared to controls (*P* < .001 for both). The QT interval and QTc difference remained present during standing without a difference in the response to standing between groups. Consequently, the ROC curves demonstrate an AUC of 0.85 (95% CI 0.78–0.92) for baseline QTc, but did not show a significant incremental diagnostic value for QTc during maximal tachycardia, QT stretching, or return to baseline (Table 2). The baseline QTc identifying LQTS patients with 90% sensitivity was 435 ms, with 65% specificity. There were no genotype differences in the response to standing (data not shown), and the inter- and intra-reader validity of the manual measurements was good to excellent for all parameters (Supplemental Table S2).

**Table 2:** Diagnostic value of QTc eventual accompanied T-wave abnormalities during the standing test

	AUC	95% CI	Cut-off @ 90% sensitivity	Specificity
*QTc*_*baseline*_	0.85	0.78-0.92	435	65%
*QTc*_*maxHR*_	0.79	0.70-0.88	476	40%
*QTc*_*stretch*_	0.80	0.72-0.89	490	62%
*QTc*_*return*_	0.82	0.73-0.89	420	44%

	AUC	95% CI	Sensitivity (95% CI)	Specificity (95% CI)
*QTc*_*baseline*_*≥440 ms*	0.73	0.65-0.81	77% (62%–88%)	70% (59%–79%)
*QTc*_*baseline*_*≥440 ms with abnormal T-waves*†	0.73	0.65-0.82	77% (62%–88%)	70% (59%–79%)
*QTc*_*stretch*_*≥490 ms*	0.75	0.68-0.82	91% (80%–98%)	58% (47%–70%)
*QTc*_*stretch*_*≥490 ms with abnormal T-waves*‡	0.73	0.67-0.80	94% (82%–99%)	53% (42%–65%)
*QTc*_*baseline*_*≥440 ms and QTc*_*stretch*_*≥490 ms*	0.69	0.62-0.78	94% (82%–99%)	44% (33%–56%)
*QTc*_*baseline*_*≥440 ms and QTc*_*stretch*_*≥490 ms with abnormal T-waves*^*§*^	0.68	0.62-0.74	96% (85%–99%)	41% (30%–52%)

AUC = area under the curve; CI = confidence interval.

† Abnormal T waves include broad, notched, and late-onset T-waves in V_4_–V_6_.

‡ Abnormal T waves include notched, biphasic, and flat T-waves in V_4_–V_6_.

T-wave morphology was not statistically different between LQTS children and controls at baseline (lead group 1, *P* = .69; lead group 2, *P* = .79; lead group 3, *P* = .74; lead group 4, *P* = 1.00). However, in response to standing, differences in T-wave patterns between LQTS children and controls arose, especially at QT stretching in lead group 3 (V_4_–V_6_, *P* = .01) and lead group 4 (I and aVL, *P* = .01) (Supplemental Figure S2). Specifically, among controls, T-wave morphology was normal in ∼97% in lead group 3 at baseline and remained normal in >70% after standing. In contrast, among LQTS children, the percentage of normal T-wave morphologies decreased from 89% at baseline to 53% after standing. At QT stretching, T-wave morphologies that best discriminated LQTS children from controls included *notched*, *biphasic*, and *flat* T waves. Therefore, we reanalyzed our results by grouping these morphologies into a single category named “*abnormal T-wave response to standing.*”

**Figure 1:**
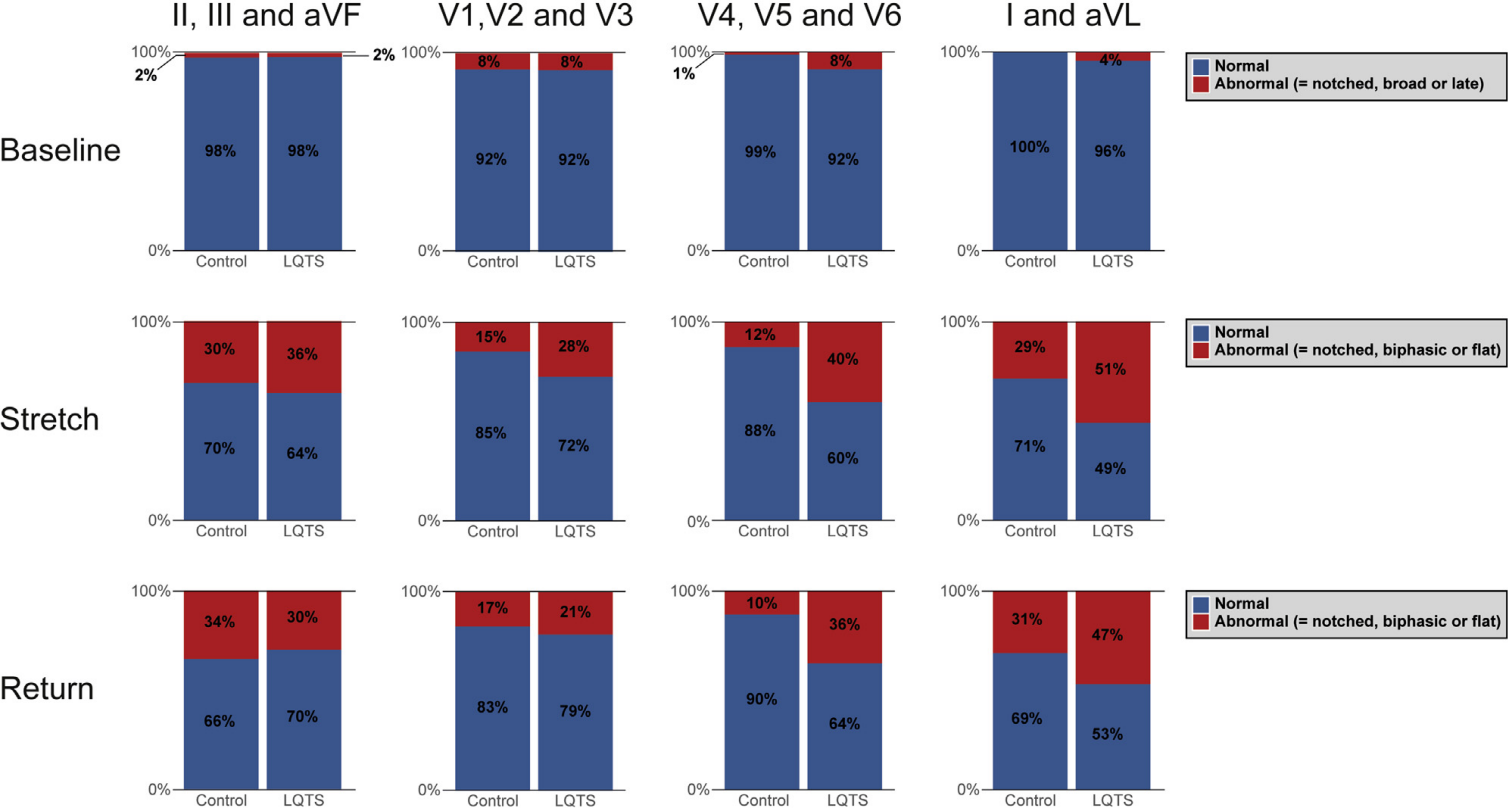
Partition of T waves at baseline and in response to standing (ie, during maximal QT stretching, and return to baseline) into “normal” and “abnormal” response in controls and long QT syndrome children for 4 different lead groups.

Figure 1 shows the partition of abnormal and normal T-wave responses. At baseline, there was no significant odds ratio for LQTS diagnosis based on the presence of abnormal T waves.^22^, ^23^, ^24^, ^25^ Hence, there was no incremental diagnostic value of the presence of T-wave abnormalities to a baseline QTc ≥440 ms (Table 2). During QT stretching, the odds ratio improved in lead groups 3 (V_4_–V_6_) and 4 (I and aVL) to an odds ratio of 5.16 (95% CI 2.14–12.43, *P* < .001) and 2.54 (95% CI 1.22–5.32, *P* = .01), respectively, for an LQTS diagnosis. Generally, for the assessment of T-wave morphology, there was a fair-to-moderate inter-reader validity and a moderate-to-substantial intra-reader validity (Supplemental Table S3).

**Figure 2:**
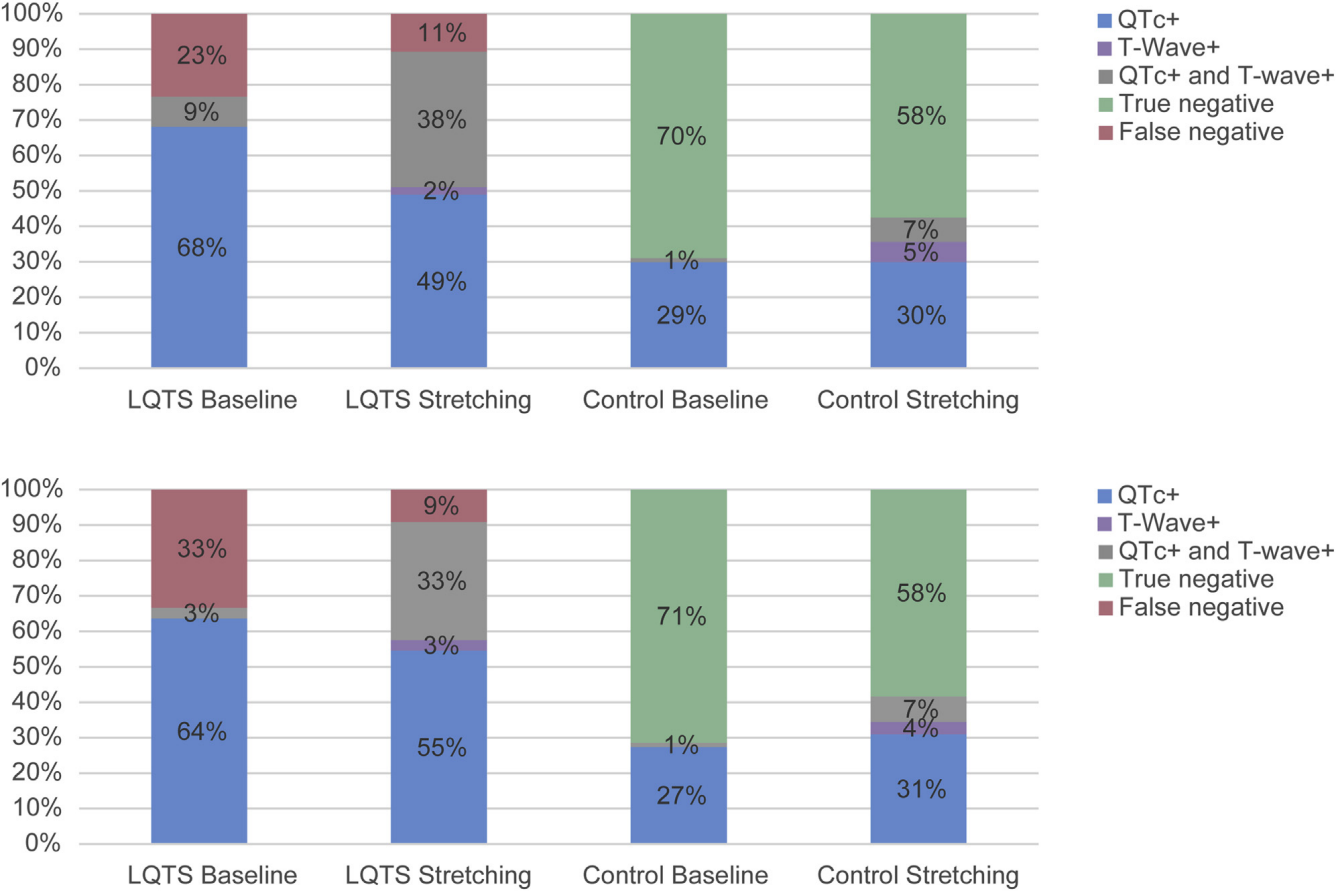
Distribution of long QT syndrome (LQTS) children and controls according to corrected QT (QTc) and T-wave morphology in leads V_4_–V_6_. **Top:** All LQTS children and controls. **Bottom:** Children without an obvious QTc prolongation at baseline (eg, <480 ms, LQTS children n = 33 and control n = 84). *At baseline,* abnormal QTc (denoted as QTc+), defined as ≥440 ms, and abnormal T waves (denoted as T-wave+) include broad, notched, and late-onset T waves. *During maximal QT stretching,* the respective abnormal values are QTc ≥490 ms and “abnormal T-wave response to standing” as defined in the text.

The incremental value of T-wave morphology assessment during QT stretching in leads V_4_–V_6_ (ie, lead group 3, *P* = .02) for an LQTS diagnosis is best appreciated from Figure 2. Although the percentage of LQTS children with an abnormal QTc increased from 77% at baseline to 87% during maximal QT stretching (absolute increment of 10%), the percentage of LQTS children who had both abnormal QTc *and* abnormal T-wave morphology had an absolute increment of 29% (from 9% at baseline to 38%). Conversely, the percentage of controls with abnormal results in both QTc and T-wave morphology increased from 1% at baseline to only 7% during maximal QT stretching. However, the sensitivity of QTc ≥490 ms during QT stretching (91%, 95% CI: 80%–98%; specificity 58%, 95% CI: 47%–70%) only slightly increased when accompanied by T-wave abnormalities (to 94%, 95% CI: 82%–99%; specificity 53%, 95% CI: 42%–65%), as shown in Table 2. This suggests that an LQTS diagnosis can be based on a QTc during QT stretching of ≥490 ms with high sensitivity and acceptable specificity, but that the diagnosis can be made with more confidence when QTc prolongation is accompanied by T-wave abnormalities. Abnormal T waves in the absence of QTc prolongation are almost as likely to represent a false-positive as a true-positive result.

When a baseline QTc ≥440 ms^3^ was accompanied by a QTc ≥490 ms and T-wave abnormalities during QT stretching, sensitivity increased to 96% (95% CI: 85%–99%; specificity 41%, 95% CI: 30%–52%). This indicates a slight additional value of the standing test as a screening test for LQTS when compared to a standard resting ECG (Table 2).

T-wave morphology changes for different LQTS genotypes are shown in Supplemental Figure S3. T-wave abnormalities provoked by standing were most helpful for diagnosing LQTS type 2 using lead group 3 (V_4_–V_6_).

#### Sensitivity analysis manual measurements

Excluding all subjects on beta-blocker therapy or including only controls and LQTS children with a baseline QTc <480 ms did not show significant differences between groups on parameters during standing or response to standing (data not shown). The incremental value for T-wave morphology assessment during QT stretching in leads V_4_–V_6_ in the subgroup with baseline QTc <480 ms was similar to the group including all LQTS children and controls (Figure 2).

### Automated measurements

A total of 71 children (53%) had an available digital standing test ECG, including 42 controls and 29 confirmed LQTS children (14 LQTS type 1, 13 LQTS type 2, and 2 LQTS type 3). Baseline characteristics, measurements at standing position, and responses to standing did not show any major differences with the total cohort (Supplemental Table S4). The inter-method validity between the automated and the manual measurements was good-to-excellent for almost all parameters (Supplemental Table S5).

#### Response to standing

**Figure 3:**
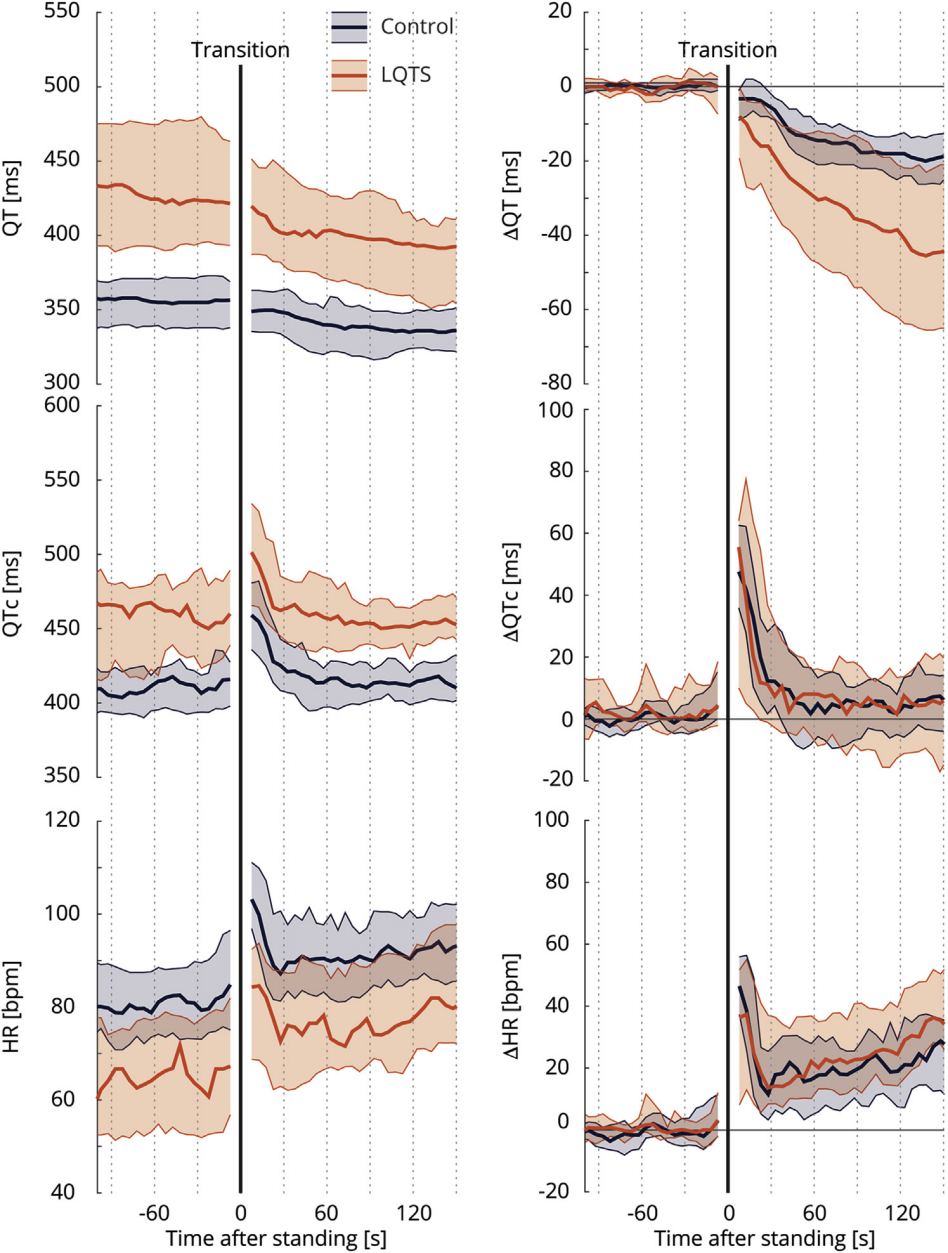
Standing test dynamics. **Left:** Median and interquartile range of *absolute* QT interval, corrected QT interval (QTc), and heart rate (HR) of controls (*blue*) and long QT syndrome (LQTS) children (*orange*). **Right:***Relative* change of QT interval, QTc, and HR to baseline values. Transition from supine to standing is indicated by the black solid line.

**Figure 4:**
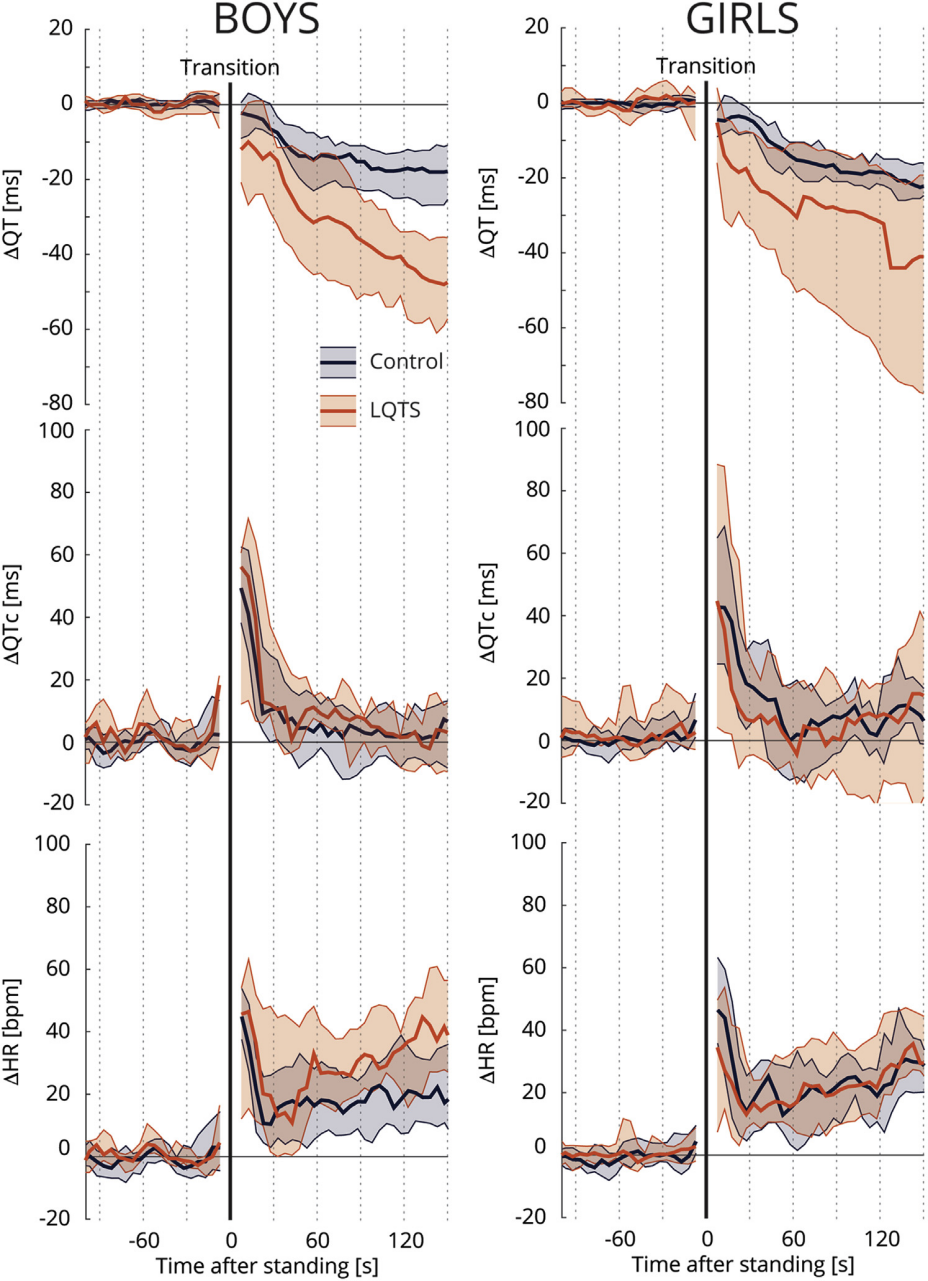
Sex difference in standing test dynamics among 36 boys (25 controls and 11 long QT syndrome [LQTS] children) and 36 girls (18 controls and 18 LQTS children). Median and interquartile ranges of *relative* changes of QT interval, corrected QT interval (QTc), and heart rate (HR) to baseline for controls (*blue*) and LQTS children (*orange*), stratified for boys (**Left column**) and girls (**Right column**). Transition from supine to standing is indicated by the black solid line.

**Figure 5:**
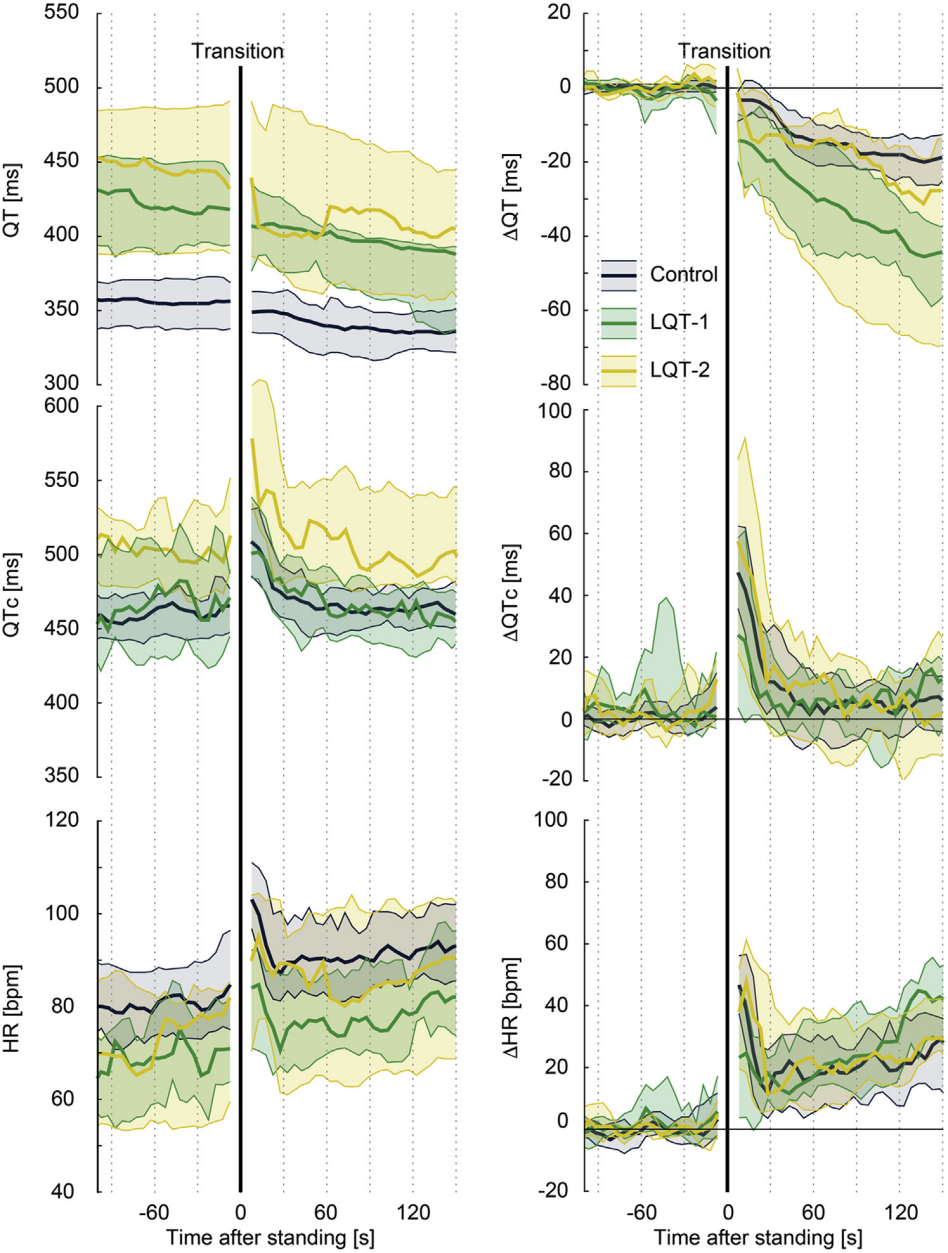
Genotype differences in standing test dynamics among 14 long QT syndrome (LQTS) type 1 (LQT-1) and 13 LQTS type 2 (LQT-2) children. The 2 LQTS type 3 children are not shown. **Left:** Median and interquartile range of *absolute* QT interval, corrected QT interval (QTc), and heart rate (HR) of controls (*blue*), LQT-1 (*green*), and LQT-2 (*yellow*). **Right:***Relative* change of QT interval, QTc, and HR to baseline values. Transition from supine to standing is indicated by the black solid line.

The beat-to-beat analyses are shown in Figure 3, 4, and 5. At baseline, LQTS children had longer QT intervals and QTc with lower heart rates compared to controls. These differences remained present after standing (Figure 3, left column). After standing up, LQTS children developed higher heart rates at about 30 seconds after standing compared to controls. While there was no difference in QTc adaptation, there was a *decrease* in absolute QT interval in LQTS children as a consequence of their *higher* heart rate upon standing. This phenomenon appeared to be more apparent in LQTS boys (Figure 4, Supplemental Figure S4) and in LQTS type 1 children (Figure 5). A sensitivity analysis including only children without beta-blocker therapy did not change these results (Supplemental Figures S5–S8).

## Discussion

We show that in our prospective cohort of children suspected for LQTS, an LQTS diagnosis can be based on a QTc during QT stretching (ie, at the incidence where the T wave end is closest to the next P wave) of ≥490 ms with a high sensitivity and acceptable specificity, and that the diagnosis can be made with more confidence when QTc prolongation is accompanied by T-wave abnormalities. However, in general, the standing test had only slight additional value as a screening test for LQTS or in the discrimination between (borderline) LQTS patients and healthy children when compared to a QTc of 440 ms on a standard resting ECG.

### Diagnostic value

The normal response to standing is a sudden acceleration of heart rate with a gradual QT interval shortening. Since the QT interval adaptation to sudden changes in heart rate is delayed (“QT hysteresis”), the vagally mediated reflex tachycardia after standing results in a transient QTc prolongation. This phenomenon is present in healthy adults,^12^ but adult LQTS patients especially fail to shorten their QT interval (impaired QT adaptation), resulting in longer QTc.^12^^,^^13^ In healthy children, however, the QT interval barely decreases after standing compared to healthy adults and, thus, QTc subsequently prolongs.^18^^,^^26^ This is mainly owing to the more important role of heart rate in children compared to adults,^17^ reflected in a decreased time to maximal heart rate and greater magnitude of heart rate changes.^12^^,^^18^

In our cohort, control children showed a slight QT interval shortening (∼10 ms) after standing compared to previous findings in healthy children (∼0 ms),^18^^,^^26^ but considerably less compared to healthy adults (∼20 ms).^12^ The observed QTc prolongation was mainly dependent on heart rate changes induced by standing. In LQTS children, there was a near-equal degree of QT interval shortening, QTc prolongation, and heart rate change compared to control children. Hence, the standing test only slightly added diagnostic value over baseline QTc and therefore this test was not very helpful in our cohort in discriminating (borderline) LQTS children and controls, in contrast to its previously shown value in adults.

T-wave morphologic changes during sudden heart rate acceleration produced by standing are valuable in an LQTS diagnosis in adults.^14^ Similar to LQTS adults,^14^ the proportion of LQTS children with normal T-wave morphology decreased to ∼30% upon standing (adults 27%, in this study 36%), while in controls T-wave morphology remained normal in >70%. But still, T-wave morphology changes appear to be of modest additional value for diagnosing LQTS in children.

### Dynamic response to standing

Standing up causes decreased *parasympathetic (vagal)* activity owing to a steep fall in blood pressure and a subsequent heart rate increase within 3 seconds. A more gradual secondary heart rate increase, around 5 seconds after stand-up, is mainly owing to further reflex inhibition of cardiac vagal tone and increased *sympathetic* outflow to the sinus node. In LQTS patients, cardiac events are often elicited by increased sympathetic activity (ie, swimming/diving or sudden loud noise)^27^ and the initiation of these arrhythmias can be suppressed by beta-adrenergic blocking agents^28^ and by denervation of the left sympathetic ganglion.^29^ It is therefore thought that LQTS patients have an autonomic nervous system imbalance, with a more prominent sympathetic tonus. It is, however, unclear whether the autonomic balance at rest or the dynamicity in autonomic activity is more relevant. We showed that LQTS children had a slightly more gradual increase in heart rate after standing up compared to controls, which can be the result of a more prominent sympathetic tonus. It is interesting to see that this phenomenon was more present in boys and LQTS type 1 children, as LQTS type 1 patients typically have cardiac events during increased sympathetic activity (eg, swimming) and LQTS type 1 boys have a higher risk during childhood and earlier onset of cardiac events than LQTS type 1 girls.^30^, ^31^, ^32^

### Limitations

This study has several limitations. First, as the study was part of regular care we were hampered with problems of “real-world data.” Particularly, the number of included children can be considered limited, and especially analyses regarding differences between LQTS subtypes were therefore hampered. However, the principal study of Viskin and colleagues^12^ included a similar cohort size (68 LQTS adults and 82 controls) and did show significant differences between LQTS adults and controls. Second, standing tests were not performed by the same investigator at a standard time of the day, nor were we informed about pretest physical activity, which could affect heart rate and repolarization,^33^ although the test was performed in a quiet setting and was only started after explanation and necessary preparations. Third, we have no data on the intra-subject variability of the test, as we did not regularly repeat the test in the same subject. And fourth, despite our prospective study, a minority of the LQTS subjects were referred after starting beta-blocker therapy. Although limited numbers prohibited further analyses into this matter, it could be that beta-blocker therapy blunted the results, though there were no indications for this based on the sensitivity analysis.

Concepts from 3 previous papers on the standing test in adults were combined in this single pediatric paper. Because results indicated limited additional value of the standing test in children over a baseline ECG, this was a negative study. Thus, it was important to include all parameters important in the adult studies. To accomplish this, the supplemental material is rather extensive.

## Conclusion

Despite promising results in adults, in children a standing test does not add significantly to the resting ECG in diagnosing LQTS in children. Thus, for children the standing test should be used with caution. This study does support further evaluation of a potential value of beat-to-beat heart rate and QT interval dynamics during the standing test in LQTS children.

## Acknowledgments

We thank Veronique M.F. Meijborg, Christian van der Werf, Louise R.A. Olde Nordkamp, and Sebastién P.J. Krul for their invaluable help in collecting ECGs for this study.

## Funding Sources

We acknowledge the support from the Netherlands CardioVascular Research Initiative: the Dutch Heart Foundation, Dutch Federation of University Medical Centres, the Netherlands Organisation for Health Research and Development, and the Royal Netherlands Academy of Sciences (PREDICT2).

## Disclosures

The authors have no conflicts to disclose.

## Authorship

All authors attest they meet the current ICMJE criteria for authorship.

## Patient Consent

Informed consent of the subjects was waived, as this study used data from regular care.

## Ethics Statement

The study was approved by the Academic Medical Center Review Board. The research reported in this paper adhered to the Helsinki Declaration as revised in 2013.

## Appendix Supplementary data

Supplemantary Material

## References

[1] Priori S.G., Blomstrom-Lundqvist C., Mazzanti A. (2015). 2015 ESC Guidelines for the management of patients with ventricular arrhythmias and the prevention of sudden cardiac death: The Task Force for the Management of Patients with Ventricular Arrhythmias and the Prevention of Sudden Cardiac Death of the European Society of Cardiology (ESC). Endorsed by: Association for European Paediatric and Congenital Cardiology (AEPC). Eur Heart J.

[2] Vincent G.M., Timothy K.W., Leppert M., Keating M. (1992). The spectrum of symptoms and QT intervals in carriers of the gene for the long-QT syndrome. N Engl J Med.

[3] Vink A.S., Neumann B., Lieve K.V.V. (2018). Determination and interpretation of the QT interval. Circulation.

[4] Moya A., Sutton R., Ammirati F. (2009). Guidelines for the diagnosis and management of syncope (version 2009). Eur Heart J.

[5] van Dijk N., Boer K.R., Colman N. (2008). High diagnostic yield and accuracy of history, physical examination, and ECG in patients with transient loss of consciousness in FAST: the Fainting Assessment study. J Cardiovasc Electrophysiol.

[6] Wilde A.A., Ackerman M.J. (2010). Exercise extreme caution when calling rare genetic variants novel arrhythmia syndrome susceptibility mutations. Heart Rhythm.

[7] Walsh R., Lahrouchi N., Tadros R. (2021). Enhancing rare variant interpretation in inherited arrhythmias through quantitative analysis of consortium disease cohorts and population controls. Genet Med.

[8] Sy R.W., van der Werf C., Chattha I.S. (2011). Derivation and validation of a simple exercise-based algorithm for prediction of genetic testing in relatives of LQTS probands. Circulation.

[9] Horner J.M., Horner M.M., Ackerman M.J. (2011). The diagnostic utility of recovery phase QTc during treadmill exercise stress testing in the evaluation of long QT syndrome. Heart Rhythm.

[10] Vyas H., Hejlik J., Ackerman M.J. (2006). Epinephrine QT stress testing in the evaluation of congenital long-QT syndrome: diagnostic accuracy of the paradoxical QT response. Circulation.

[11] Shimizu W., Noda T., Takaki H. (2004). Diagnostic value of epinephrine test for genotyping LQT1, LQT2, and LQT3 forms of congenital long QT syndrome. Heart Rhythm.

[12] Viskin S., Postema P.G., Bhuiyan Z.A. (2010). The response of the QT interval to the brief tachycardia provoked by standing: a bedside test for diagnosing long QT syndrome. J Am Coll Cardiol.

[13] Adler A., van der Werf C., Postema P.G. (2012). The phenomenon of "QT stunning": the abnormal QT prolongation provoked by standing persists even as the heart rate returns to normal in patients with long QT syndrome. Heart Rhythm.

[14] Chorin E., Havakuk O., Adler A. (2015). Diagnostic value of T-wave morphology changes during "QT stretching" in patients with long QT syndrome. Heart Rhythm.

[15] Zareba W., Moss A.J. (2001). Long QT syndrome in children. J Electrocardiol.

[16] Ganzeboom K.S., Colman N., Reitsma J.B., Shen W.K., Wieling W. (2003). Prevalence and triggers of syncope in medical students. Am J Cardiol.

[17] Ives C.T., Kimpinski K. (2013). Higher postural heart rate increments on head-up tilt correlate with younger age but not orthostatic symptoms. J Appl Physiol (1985).

[18] Filippini L., Postema P.G., Zoubin K. (2018). The brisk-standing-test for long QT syndrome in prepubertal school children: defining normal. Europace.

[19] Bazett (1920). An analysis of the time relationship of electrocardiograms. Heart Rhythm.

[20] Hermans B.J.M., Vink A.S., Bennis F.C. (2017). The development and validation of an easy to use automatic QT-interval algorithm. PLoS One.

[21] DeLong E.R., DeLong D.M., Clarke-Pearson D.L. (1988). Comparing the areas under two or more correlated receiver operating characteristic curves: a nonparametric approach. Biometrics.

[22] Moss A.J., Zareba W., Benhorin J. (1995). ECG T-wave patterns in genetically distinct forms of the hereditary long QT syndrome. Circulation.

[23] Zhang L., Timothy K.W., Vincent G.M. (2000). Spectrum of ST-T-wave patterns and repolarization parameters in congenital long-QT syndrome: ECG findings identify genotypes. Circulation.

[24] Lehmann M.H., Suzuki F., Fromm B.S. (1994). T wave "humps" as a potential electrocardiographic marker of the long QT syndrome. J Am Coll Cardiol.

[25] Dausse E., Berthet M., Denjoy I. (1996). A mutation in HERG associated with notched T waves in long QT syndrome. J Mol Cell Cardiol.

[26] Dionne A., Fournier A., Dahdah N., Abrams D., Khairy P., Abadir S. (2018). Dynamic QT interval changes from supine to standing in healthy children. Can J Cardiol.

[27] Moss A.J., Schwartz P.J., Crampton R.S. (1991). The long QT syndrome. Prospective longitudinal study of 328 families. Circulation.

[28] Priori S.G., Napolitano C., Schwartz P.J. (2004). Association of long QT syndrome loci and cardiac events among patients treated with beta-blockers. JAMA.

[29] Schwartz P.J., Priori S.G., Cerrone M. (2004). Left cardiac sympathetic denervation in the management of high-risk patients affected by the long-QT syndrome. Circulation.

[30] Costa J., Lopes C.M., Barsheshet A. (2012). Combined assessment of sex- and mutation-specific information for risk stratification in type 1 long QT syndrome. Heart Rhythm.

[31] Locati E.H., Zareba W., Moss A.J. (1998). Age- and sex-related differences in clinical manifestations in patients with congenital long-QT syndrome: findings from the International LQTS Registry. Circulation.

[32] Zareba W., Moss A.J., Locati E.H. (2003). Modulating effects of age and gender on the clinical course of long QT syndrome by genotype. J Am Coll Cardiol.

[33] Porta A., Girardengo G., Bari V. (2015). Autonomic control of heart rate and QT interval variability influences arrhythmic risk in long QT syndrome type 1. J Am Coll Cardiol.

