# Supplementary material for: Diagnostic accuracy of the response to the brief tachycardia provoked by standing in children suspected for long QT syndrome
Source: Heart Rhythm O2. 2021 Mar 13;2(2):149–59. doi: 10.1016/j.hroo.2021.03.005 (PMC8183857; doi:10.1016/j.hroo.2021.03.005)
Supplement: Supplemantary Material [file mmc1.pdf]

Supplementary material

Supplementary Tables

	Control<br>n=86	LQTS<br>n=47	Possible LQTS<br>n=15	Other<br>n=11	p-value
Age, years	10 (7-14)	12 (8-15)	13 (10-15)	13 (12-15)	0.085
Girls	39 (45%)	29 (62%)	11 (73%)	2 (18%)	0.012
Presentation					**<0.001**
Family screening	47 (55%)	38 (81%)	3 (20%)	1 (9%)	
Family SCD	8 (9%)	0 (0%)	1 (7%)	2 (18%)	
Near-drowning/OHCA/ACA	1 (1%)	0 (0%)	2 (13%)	3 (27%)	
Other	30 (35%)	9 (19%)	9 (60%)	5 (45%)	
Symptomatic at presentation	1 (1%)	3 (6%)	2 (13%)	3 (27%)	0.002
BB-therapy	1 (1%)	9 (19%)	4 (27%)	2 (18%)	**<0.001**
Supine position¥					
$HR_{baseline}$,bpm	81 (±15)	73 (±16)	74 (±17)	71 (±13)	0.028
$QT_{baseline}$,ms	367 (±34)	429 (±58)	415 (±60)	378 (±30)	**<0.001**
$QTc_{baseline}$	421 (±29)	466 (±36)	452 (±39)	410 (±28)	**<0.001**
Standing position¥					
HR_{maxHR},bpm	112 (±15)	100 (±17)	110 (±19)	102 (±14)	**<0.001**
QT_{maxHR},ms	360 (±34)	421 (±60)	393 (±56)	374 (±44)	**<0.001**
QTc_{maxHR},ms	489 (±37)	537 (±51)	526 (±55)	483 (±37)	**<0.001**
$HR_{stretch}$,bpm	110 (±15)	99 (±17)	109 (±20)	99 (±11)	**0.001**
$QT_{stretch}$,ms	363 (±36)	429 (±62)	395 (±55)	378 (±46)	**<0.001**
$QTc_{stretch}$,ms	489 (±42)	544 (±56)	527 (±55)	483 (±40)	**<0.001**
QT_{return},ms	371 (±39)	450 (±74)	405 (±61)	376 (±26)	**<0.001**
QTc_{return},ms	429 (±38)	492 (±60)	456 (±70)	406 (±35)	**<0.001**
Response to standing¥					
Time to maximal tachycardia,s	11 (9-14)	11 (10-13)	11 (8-13)	9 (7-12)	0.222
Time to maximal QT-stretching,s	11 (9-14)	10 (9-12)	10 (8-13)	8 (7-11)	0.293
Time to return to baseline,s	21 (18-27)	20 (19-29)	20 (17-29)	21 (17-23)	0.902
ΔHR during maximal tachycardia,bpm	32 (±11)	27 (± 9)	36 (± 9)	30 (±11)	0.008
ΔQT during maximal tachycardia,ms	-9 (±22)	-8 (±30)	-22 (±35)	-3 (±29)	0.269
ΔQTc during maximal tachycardia,ms	67 (±41)	71 (±47)	73 (±47)	75 (±49)	0.908
ΔHR during maximal QT-stretching,bpm	31 (±11)	26 (± 9)	35 (± 9)	28 (± 6)	0.009
ΔQT during maximal QT-stretching,ms	-7 (±21)	0 (±39)	-20 (±40)	1 (±34)	0.145
ΔQTc during maximal QT-stretching,ms	67 (±43)	78 (±54)	74 (±53)	74 (±51)	0.658
ΔQT upon return to baseline HR,ms	5 (±28)	22 (±47)	-5 (±58)	2 (±23)	0.062
ΔQTc upon return to baseline HR,ms	8 (±31)	26 (±50)	-1 (±70)	5 (±22)	0.067

Supplementary Table S1. Baseline characteristics and manual ECG-measurements. Differences between groups were tested using a χ2-test for categorical variables, and with a one–way ANOVA or Kruskal Wallis test for continuous variables as appropriate. For post-hoc analyses a Bonferroni correction was used in order to correct for multiple comparisons. **¥** p-values <0.002 are considered to be significant.
SCD=Sudden Cardiac Death, OHCA=Out of Hospital Cardiac Arrest, ACA=Aborted Cardiac Arrest, BB=beta-blocker, HR=heart rate, QTc=QT-interval corrected for heart rate using Bazett’s formula, bpm=beats per minute, (m)s=(milli)seconds.

	Inter-reader		*Intra-reader*	
	ICC (95% CI)	*Mean (±95% LoA)*	*ICC (95% CI)*	*Mean (±95% LoA)*
Supine position				
$HR_{baseline}$,bpm	0.807 (0.378-0.938)	6 (± 15)	0.843 (0.605-0.994)	3 (±16)
$QT_{baseline}$,ms	0.919 (0.779-0.972)	4 (± 66)	0.870 (0.614-0.956)	17 (±57)
Standing position				
HR_{maxHR},bpm	0.937 (0.822-0.978)	0 (± 11)	0.904 (0.772-0.968)	3 (±12)
QT_{maxHR},ms	0.939 (0.820-0.979)	9 (± 40)	0.885 (0.683-0.962)	4 (±58)
$HR_{stretch}$,bpm	0.935 (0.813-0.979)	1 (± 12)	0.920 (0.778-0.973)	2 (±13)
$QT_{stretch}$,ms	0.916 (0.757-0.972)	0 (± 51)	0.902 (0.723-0.967)	4 (±60)
HR_{return},bpm	0.832 (0.478-0.945)	5 (± 15)	0.843 (0.597-0.944)	4 (±16)
QT_{return},ms	0.784 (0.464-0.922)	3 (±121)	0.853 (0.618-0.948)	17 (±78)

Supplementary Table S2. Inter- and intra-reader validity.
To determine inter- and intra-reader measurement validity, a random sample of 10% was measured by an additional reader (JP) and re-measured by the principal reader (SV). Inter- and intra-reader were expressed as the intra-class correlation coefficient (ICC) for single measurements for continuous variables based on a two-way agreement (inter-reader validity) and consistency (intra-reader validity) model according to Cicchetti[1] and Fleiss[2]. Bland-Altman analyses[3] were then performed to assess bias and 95% limits of agreement (LoA).
HR=heart rate, QTc=QT-interval corrected for heart rate using Bazett's formula, bpm=beats per minute, ms=milliseconds, IC=confidence interval.

1. Cicchetti DV, Sparrow SA. Developing criteria for establishing interrater reliability of specific items: applications to assessment of adaptive behavior. American journal of mental deficiency Sep 1981;86:127-137.
2. Fleiss JL. Statistical methods for rates and proportions. New York, N.Y: John Wiley and Sons; 1981.
3. Bland JM, Altman DG. Comparing methods of measurement: why plotting difference against standard method is misleading. Lancet (London, England) Oct 21 1995;346:1085-1087.

	Inter-reader		Intra-reader	
	Agreement	*Kappa*	*Agreement*	*Kappa*
Baseline				
Lead group I	80%	0.21	87%	0.61
Lead group II	67%	0.53	80%	0.68
Lead group III	87%	0.30	93%	0.64
Lead group IV	60%	0.22	87%	0.75
QT-stretch				
Lead group I	27%	0.07	73%	0.58
Lead group II	47%	0.31	67%	0.52
Lead group III	67%	0.53	100%	1.00
Lead group IV	33%	0.13	87%	0.80
Return				
Lead group I	47%	0.23	67%	0.46
Lead group II	67%	0.53	80%	0.71
Lead group III	60%	0.20	87%	0.75
Lead group IV	73%	0.54	93%	0.89

Supplementary Table S3. Inter- and intra-reader validity for T-wave morphology expressed as cohen's kappa statistic from a random sample of 10% that was measured by an additional reader (JP) and re-measured by the principal reader (SV).
Lead group I = II, III, aVF, Lead group II = V1-V3, Lead group II = V4-V6, Lead group II = I and aVL.

	Control<br>n=42	LQTS<br>n=29	Possible LQTS<br>n=10	Other<br>n=3	p-value
Age, years	9 (7-14)	13 (10-15)	14 (11-17)	16 (14-16)	0.021
Girls	18 (43%)	18 (62%)	6 (60%)	1 (33%)	0.357
Presentation					**<0.001**
Family screening	18 (43%)	25 (86%)	2 (20%)	0 (0%)	
Family SCD	1 (2%)	0 (0%)	0 (0%)	0 (0%)	
Near-drowning/OHCA/ACA	1 (2%)	0 (0%)	2 (20%)	1 (33%)	
Other	22 (52%)	4 (14%)	6 (60%)	2 (67%)	
Symptomatic at presentation	1 (2%)	2 (9%)	2 (20%)	1 (33%)	0.046
BB-therapy	1 (2%)	6 (21%)	3 (30%)	0 (0%)	0.014
Supine position¥					
$HR_{baseline}$,bpm	78 (±12)	69 (±16)	72 (±20)	59 (± 6)	0.030
$QT_{baseline}$,ms	356 (±27)	433(±56)	381 (±57)	375 (±30)	**<0.001**
$QTc_{baseline}$,ms	402 (±28)	455 (±36)	405 (±27)	373 (±49)	**<0.001**
Standing position¥					
HR_{maxHR},bpm	115 (±13)	99 (±15)	111(±23)	91 (± 4)	**<0.001**
QT_{maxHR},ms	347 (±32)	421 (±56)	381 (±51)	373 (±28)	**<0.001**
QTc_{maxHR},ms	479 (±34)	536 (±45)	509 (±50)	459 (±28)	**<0.001**
$HR_{stretch}$,bpm	113 (±14)	97 (±16)	109 (±23)	90 (± 3)	**<0.001**
$QT_{stretch}$,ms	355 (±31)	429 (±57)	395 (±51)	372 (±30)	**<0.001**
$QTc_{stretch}$,ms	486 (±35)	539 (±43)	526 (±54)	456 (±32)	**<0.001**
QT_{return},ms	359 (±33)	432 (±49)	390 (±49)	373 (±29)	**<0.001**
QTc_{return},ms	406 (±44)	454 (±43)	427 (±41)	372 (±47)	**<0.001**
Response to standing¥					
Time to maximal tachycardia,s	11 (10-14)	12 (11-15)	11 (8-14)	8 (8-10)	0.445
Time to maximal QT-stretching,s	11 (9-13)	10 (9-13)	11 (9-13)	7 (7-10)	0.692
Time to return to baseline,s	26 (22-28)	24 (21-28)	25 (23-27)	20 (17-23)	0.624
ΔHR during maximal tachycardia,bpm	38 (±12)	30 (± 9)	39 (±13)	32 (± 8)	0.031
ΔQT during maximal tachycardia,ms	-8 (±23)	-11 (±26)	-1 (±22)	-2 (±10)	0.616
ΔQTc during maximal tachycardia,ms	77 (±36)	80 (±39)	104 (±49)	86 (±23)	0.254
ΔHR during maximal QT-stretching,bpm	36 (±12)	28 (± 9)	38 (±13)	31 (± 7)	0.025
ΔQT during maximal QT-stretching,ms	-1 (±17)	-3 (±25)	14 (±26)	-3 (± 8)	0.167
ΔQTc during maximal QT-stretching,ms	83 (±33)	83 (±39)	120 (±51)	82 (±19)	0.042
ΔQT upon return to baseline HR,ms	4 (±17)	0 (±25)	9 (±22)	-2 (± 6)	0.599
ΔQTc upon return to baseline HR,ms	4 (±30)	-2 (±34)	22 (±31)	-2 (±12)	0.247

Supplementary Table S4. Baseline characteristics and automatic ECG-measurements. Differences between groups were tested using a χ2-test for categorical variables, and with a one–way ANOVA or Kruskal Wallis test for continuous variables as appropriate. For post-hoc analyses a Bonferroni correction was used in order to correct for multiple comparisons. **¥** p-values <0.002 are considered to be significant.
HR=heart rate, QTc=QT-interval corrected for heart rate using Bazett's formula, bpm=beats per minute, (m)s=(milli)seconds.

	Inter-method	
	ICC (95% CI)	*Mean (±95% LoA)*
Supine position		
$HR_{baseline}$,bpm	0.915 (0.872-0.944)	3 (± 12)
$QT_{baseline}$,ms	0.898 (0.847-0.933)	12 (± 50)
Standing position		
$HR_{maxHR,}$bpm	0.980 (0.969-0.987)	1 (± 7)
QT_{maxHR},ms	0.903 (0.854-0.936)	9 (± 49)
$HR_{stretch,}$ bpm	0.969 (0.953-0.980)	0 (± 9)
$QT_{stretch}$,ms	0.873 (0.811-0.916)	5 (± 58)
$HR_{return,}$bpm	0.845 (0.768-0.898)	4 (± 17)
QT_{return},ms	0.774 (0.667-0.850)	20 (± 84)

Supplementary Table S5. Inter-method validity expressed as the intra-class correlation coefficient (ICC) for single measurements for continuous variables based on a two-way consistency model according to Cicchetti[1] and Fleiss[2]. Bland-Altman analyses[3] were then performed to assess bias and 95% limits of agreement (LoA).
HR=heart rate, QTc=QT-interval corrected for heart rate using Bazett's formula, bpm=beats per minute, ms=milliseconds, IC=confidence interval.

1. Cicchetti DV, Sparrow SA. Developing criteria for establishing interrater reliability of specific items: applications to assessment of adaptive behavior. American journal of mental deficiency Sep 1981;86:127-137.
2. Fleiss JL. Statistical methods for rates and proportions. New York, N.Y: John Wiley and Sons; 1981.
3. Bland JM, Altman DG. Comparing methods of measurement: why plotting difference against standard method is misleading. Lancet (London, England) Oct 21 1995;346:1085-1087.

Supplementary Figures

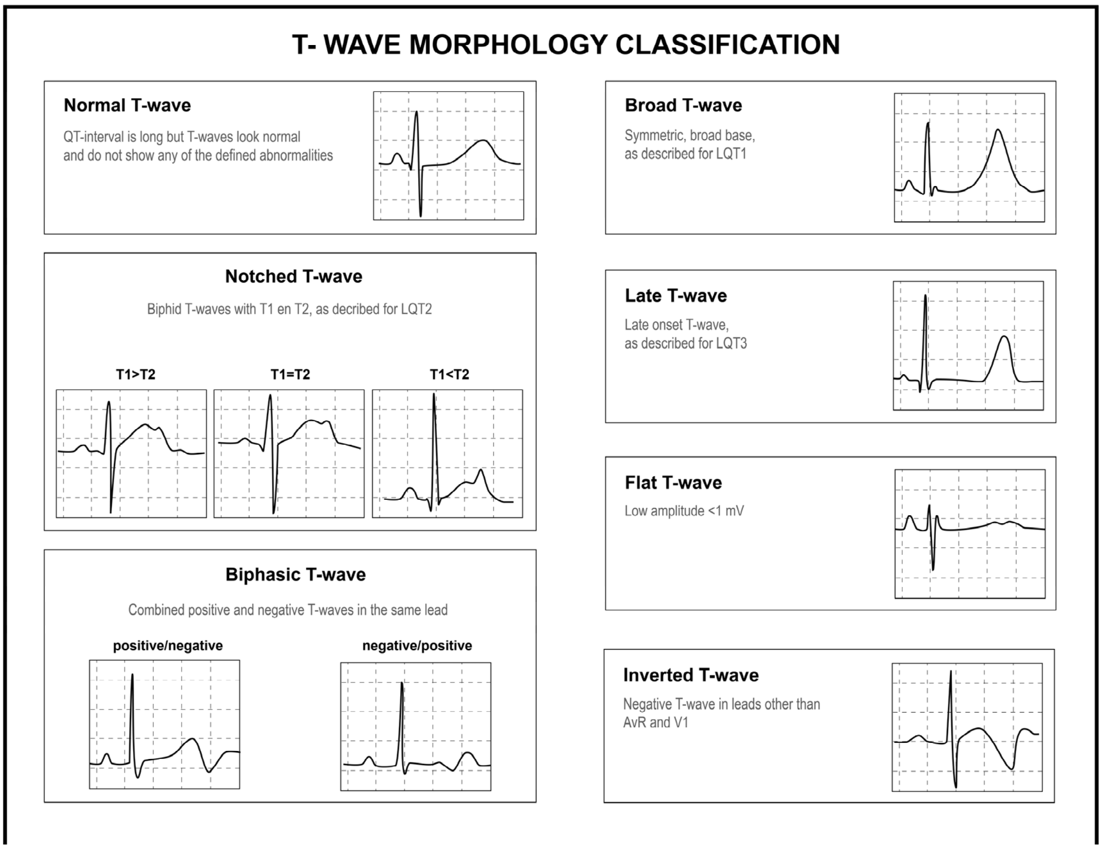

Supplementary Figure S1. T-wave morphology classification adapted from Chorin et al.[14]

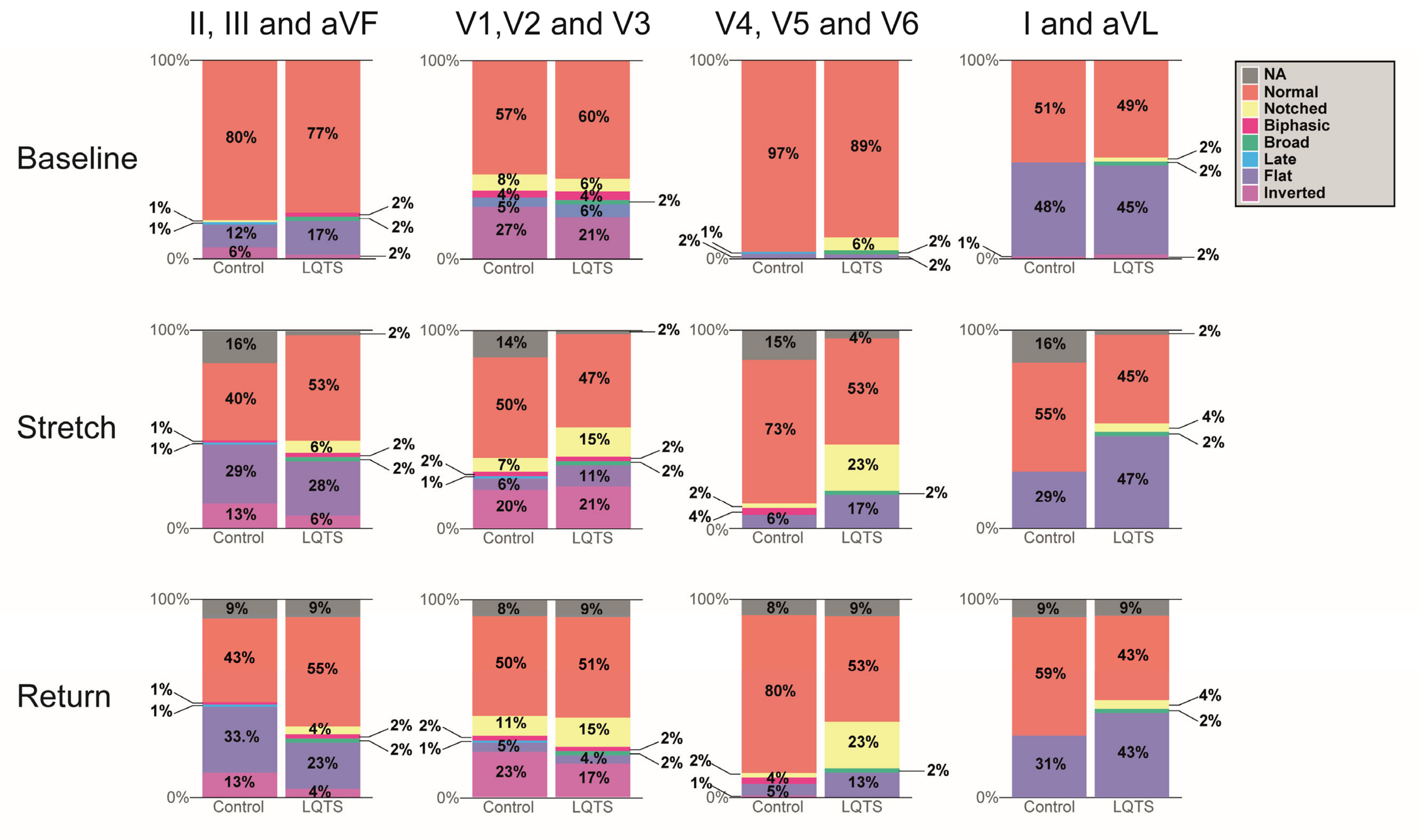

Supplementary Figure S2. Percentage of present T-wave morphologies at three phases of the standing-test (i.e. at baseline, during maximal QT-stretching, and return to baseline) in controls and LQTS-children for four different lead-groups.

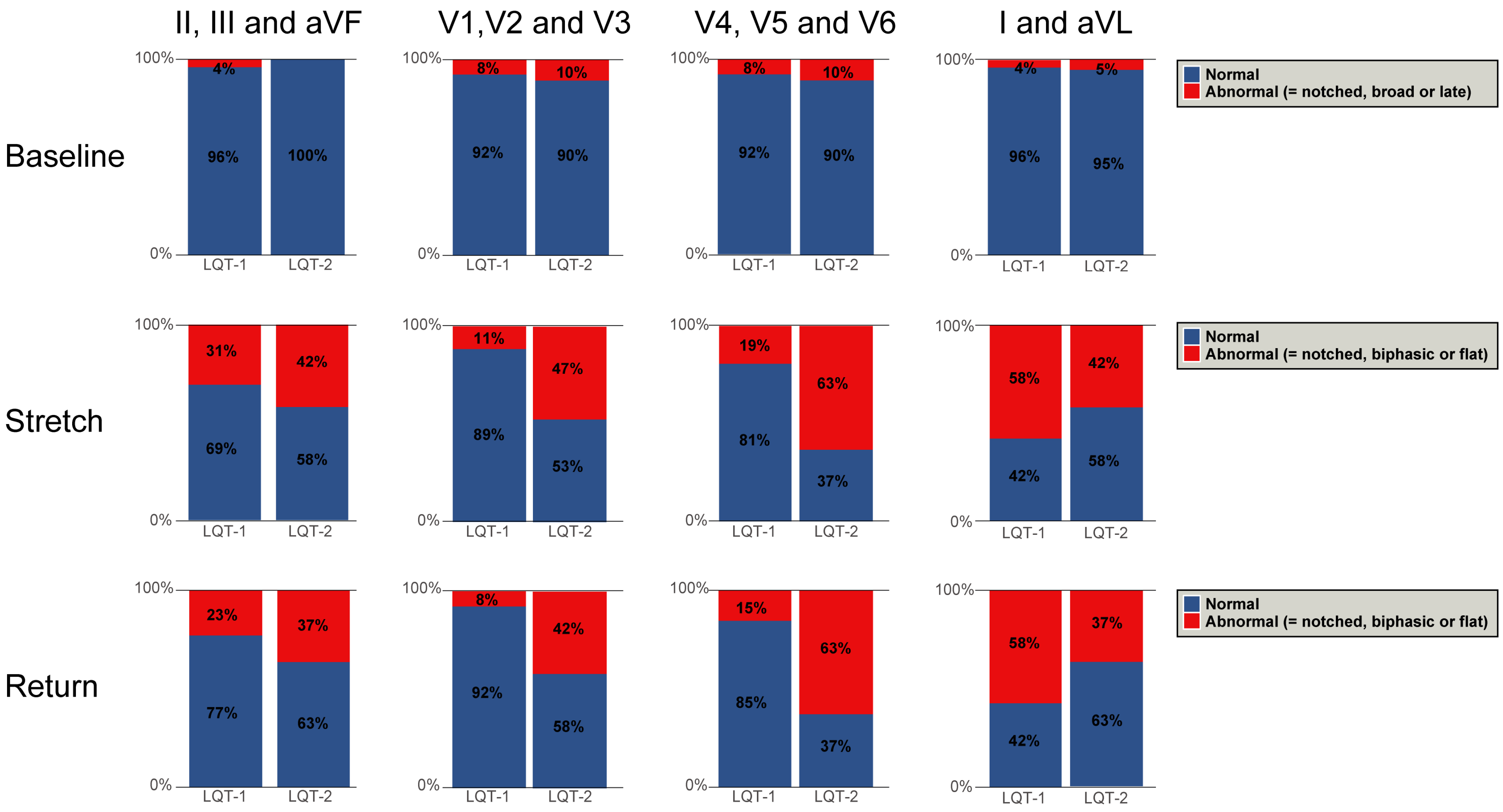

Supplementary Figure S3. Partition of T-waves at baseline and in response to standing into “normal” and “abnormal” response in LQTS-type-1 (LQT-1, n=26) and LQTS-type-2 (LQT-2, n=19). The two LQTS-type-3-patients are not shown.

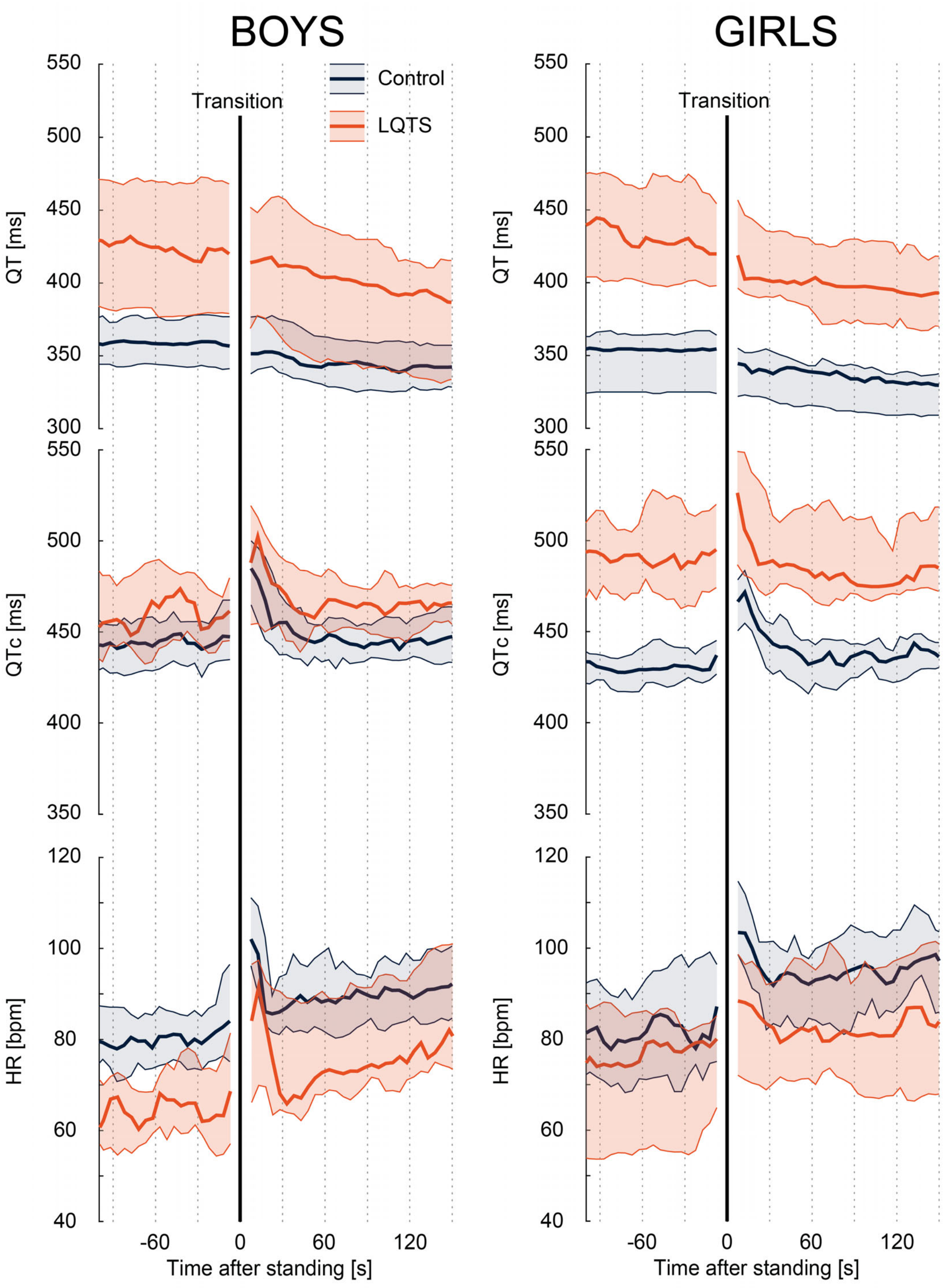

Supplementary Figure S4. Sex-difference in standing-test dynamics. Including 36 boys (25 controls and 11 LQTS-children) and 36 girls (18 controls and 18 LQTS-children). The median and interquartile range of the absolute QT-interval, QTc and heart rate of controls (blue) and LQTS-patients (orange), stratified for boys (**left column**) and girls (**right column**). Transition from supine to standing is indicated by the black solid line. HR=heart rate, LQTS=Long QT-syndrome, QTc=QT-interval corrected for heart rate using Bazett's formula.

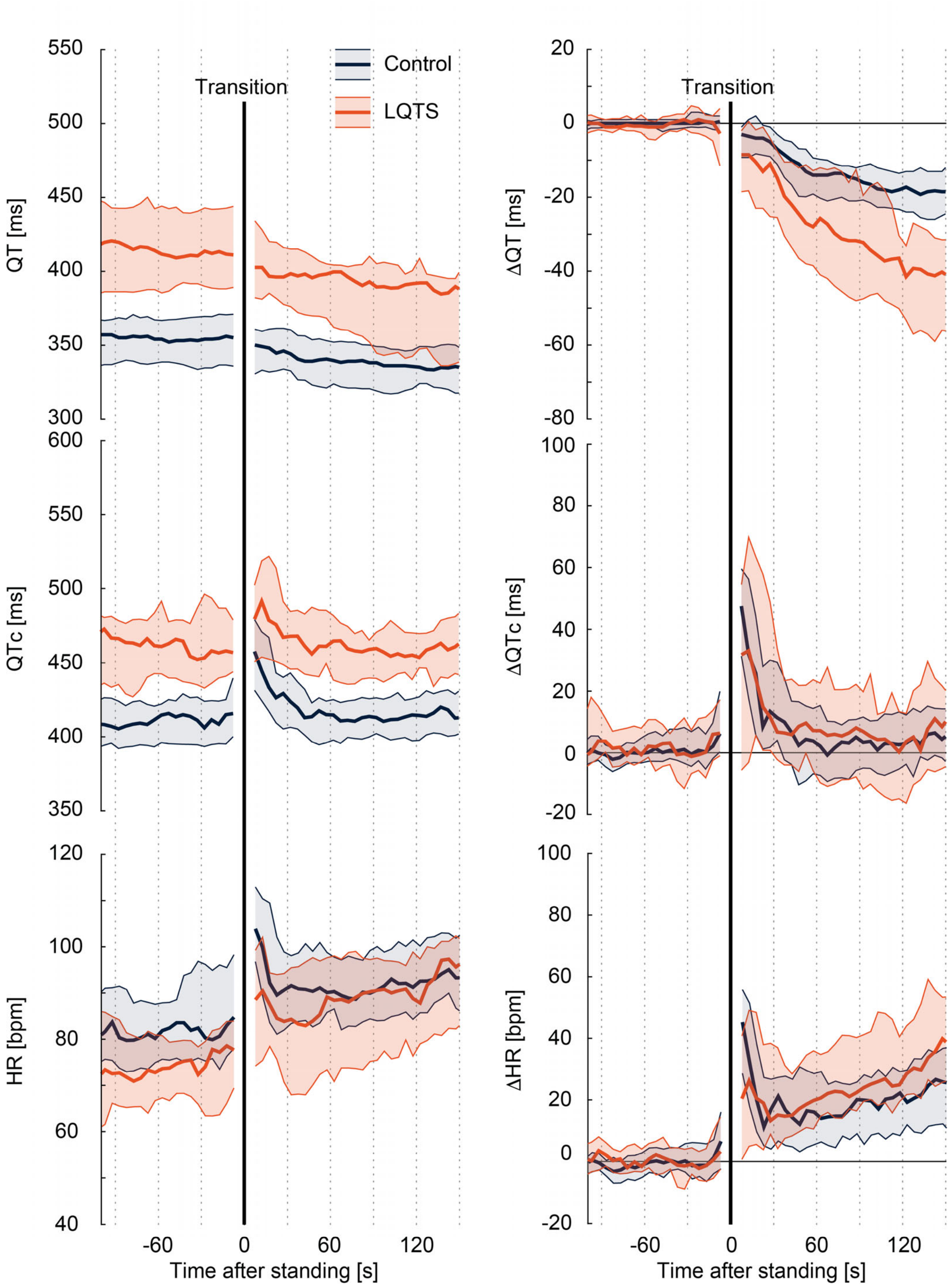

Supplementary Figure S5. Standing-test dynamics in controls (n=41) and LQTS-patients (n=21) without beta-blocker therapy. **Left**: median and interquartile range of the absolute QT-interval, QTc and heart rate of controls (blue) and LQTS-patients (orange). **Right**: relative change of the QT-interval, QTc and heart rate to the baseline values for controls and LQTS-patients. Transition from supine to standing is indicated by the black solid line. HR=heart rate, LQTS=Long QT-syndrome, QTc=QT-interval corrected for heart rate using Bazett's formula.

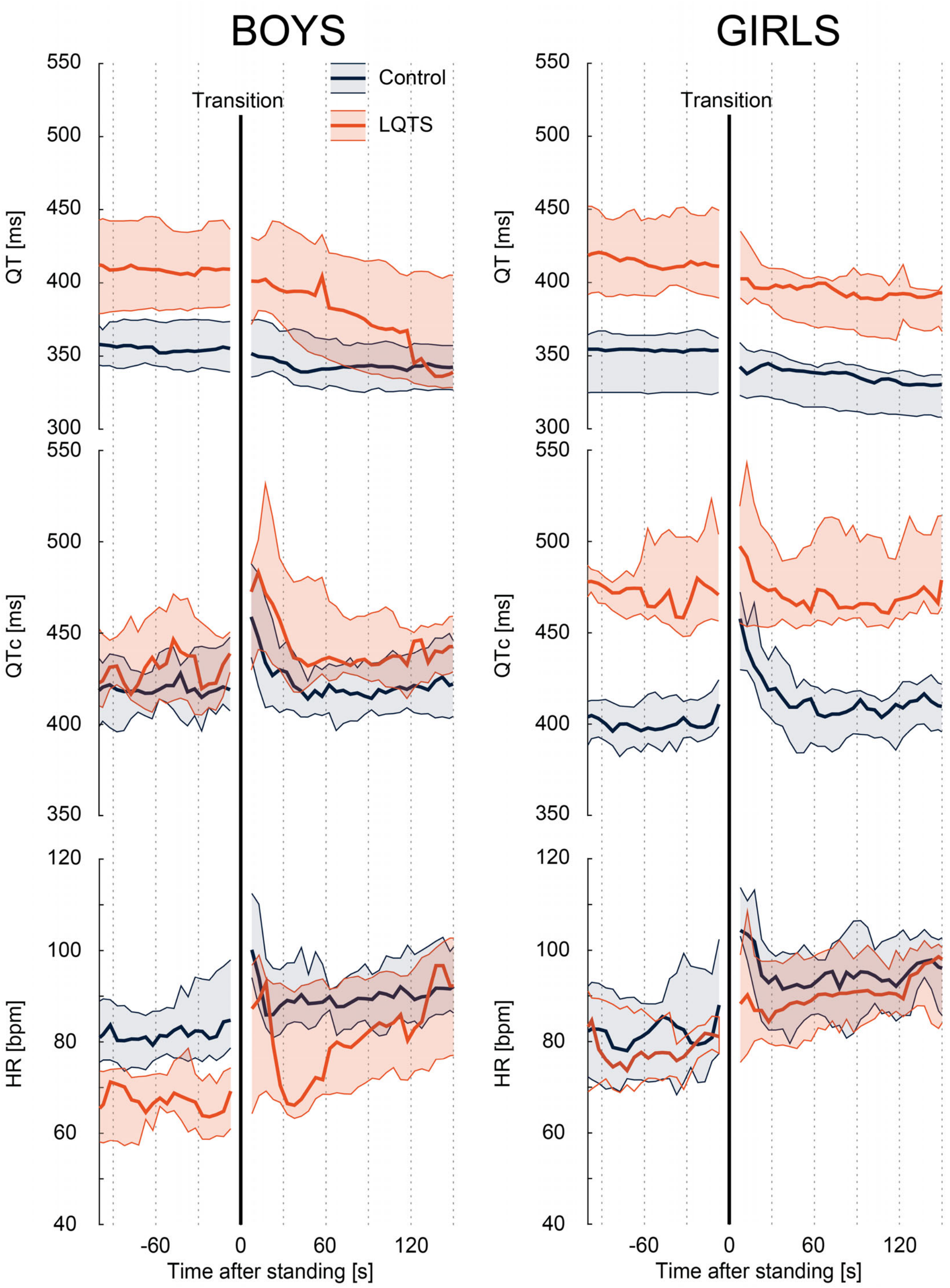

Supplementary Figure S6. Sex-difference in standing-test dynamics in controls and LQTS-patients without beta-blocker therapy. Including 31 boys (23 controls and 8 LQTS-patients) and 31 girls (18 controls and 13 LQTS-patients). The median and interquartile range of the absolute QT-interval, QTc and heart rate of controls (blue) and LQTS-patients (orange), stratified for boys (**left column**) and girls (**right column**). Transition from supine to standing is indicated by the black solid line. HR=heart rate, LQTS=Long QT-syndrome, QTc=QT-interval corrected for heart rate using Bazett's formula.

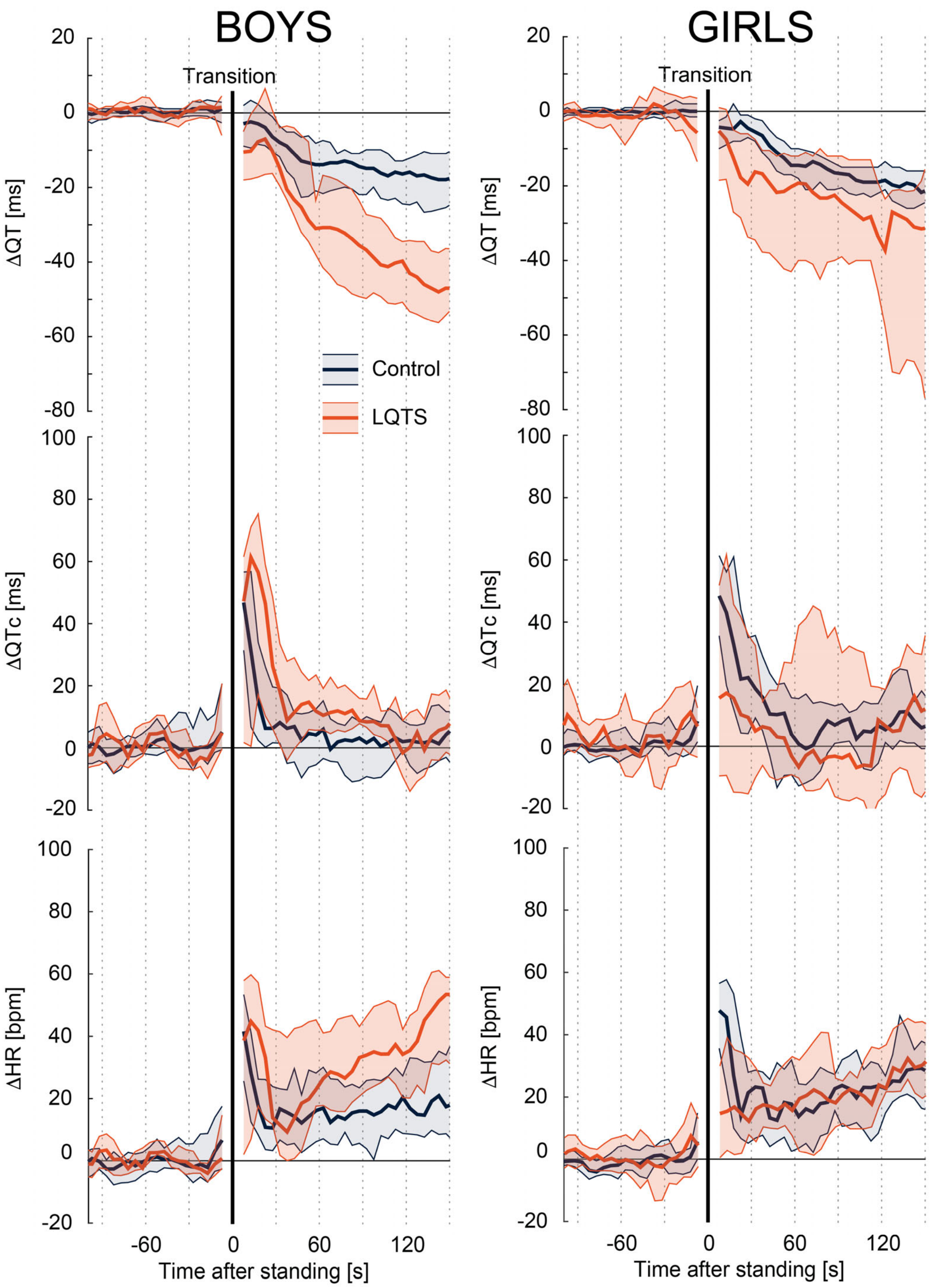

Supplementary Figure S7. Sex-difference in standing-test dynamics in controls and LQTS-patients without beta-blocker therapy. Including 31 boys (23 controls and 8 LQTS-patients) and 31 girls (18 controls and 13 LQTS-patients). The median and interquartile ranges of the relative change of the QT-interval, QTc and heart rate to the baseline for controls (blue) and LQTS-patients (orange), stratified for boys (**left column**) and girls (**right column**). Transition from supine to standing is indicated by the black solid line. HR=heart rate, LQTS=Long QT-syndrome, QTc=QT-interval corrected for heart rate using Bazett's formula.

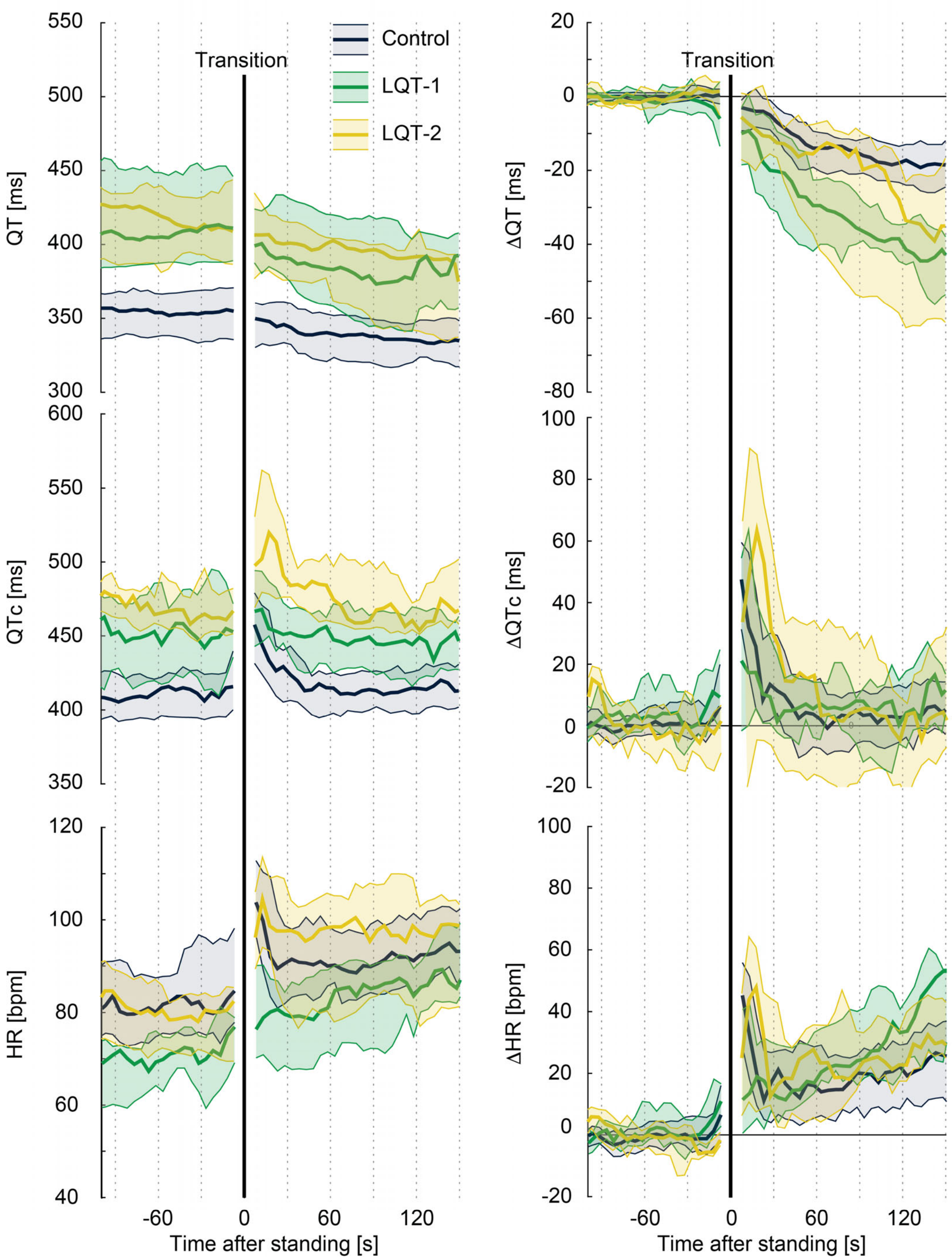

Supplementary Figure S8. Genotype-differences in standing-test dynamics in controls and LQTS-patients without beta-blocker therapy. Including 12 LQT-1 and 8 LQT-2 patients. **Left**: median and interquartile range of the absolute QT-interval, QTc and heart rate of controls (blue), LQT-1-patients (green) and LQT-2-patients (yellow). **Right**: relative change of the QT-interval, QTc and heart rate to the baseline values for controls and LQTS-patients. Transition from supine to standing is indicated by the black solid line. HR=heart rate, LQTS=Long QT-syndrome, QTc=QT-interval corrected for heart rate using Bazett's formula.
